# An audit of immunofixation requesting practices at a South African referral laboratory

**DOI:** 10.4102/ajlm.v3i1.91

**Published:** 2014-10-08

**Authors:** Verena Gounden, Yashna Rampursat

**Affiliations:** 1 Department of Chemical Pathology, National Health Laboratory Services, Inkosi Albert Luthuli Central Hospital, South Africa; 2 Nelson R Mandela School of Medicine, University of KwaZulu-Natal, South Africa

## Abstract

**Background:**

It is common practice in most chemical pathology laboratories for reflective immunofixation electrophoresis (IFE) to occur following the detection or suspicion of a paraprotein on serum protein electrophoresis (SPEP). The chemical pathology laboratory at Inkosi Albert Luthuli Central Hospital (IALCH) in Durban, South Africa, is currently the only non-private laboratory in the KwaZulu Natal province that performs SPEP analysis, with current practice requiring that the clinician request IFE following suggestion by the laboratory after a suspicious SPEP result.

**Objectives:**

To review the current process for IFE at IALCH in the context of reflective testing and to examine the use of the alpha-2-globulin/alpha-1-globulin ratio as a predictor of a positive IFE result.

**Methods:**

Data for 1260 consecutive SPEP tests performed at the IALCH National Health Laboratory Service were collected between February and July 2011. SPEP and IFE were performed with a Sebia Hydrasys automated electrophoresis system. The alpha-2-globulin/alpha-1-globulin ratio was calculated using density of corresponding fractions on SPEP.

**Results:**

Analysis revealed that of the 1260 SPEPs performed during the analysis period, 304 IFEs were suggested by the reviewing pathologist. A total of 45 (15%) of the suggested IFEs were subsequently requested by the attending clinicians. Almost half (46.5%) (*n* = 20) of the suggested IFEs that were performed revealed the presence of a paraprotein. There was no statistically-significant difference between the alpha-2-globulin/alpha-1-globulin ratio for patients with positive or negative IFEs (*p*-value = 0.2).

**Conclusions:**

This study reveals the need for reflective addition of IFE testing by the laboratory following suspicious findings on SPEP.

## Introduction

Overproduction of a single abnormal clone of a plasma cell or B lymphocyte results in the presence of a monoclonal gammopathy.^1^ Disorders associated with the presence of a monoclonal protein (M protein) include B-cell lymphomas and leukaemias; amyloidosis and Waldenstrom’s macroglobulinaemia; and plasma cell dyscrasias, which include multiple myeloma, monoclonal gammopathy of undetermined significance (MGUS) and plasmacytoma.^2^ MGUS is a premalignant plasma cell disorder with an associated risk of progression to multiple myeloma (MM). MM is currently recognised as being largely incurable; the ability to identify the premalignant condition (MGUS) is important for strategies that attempt to delay or prevent progression to MM. It is also important to distinguish between those patients with MM and MGUS as the management of these two groups of patients differs, with those with MGUS being treated conservatively.^1^

The M protein is identified by either serum protein electrophoresis (SPEP) or urine protein electrophoresis (UPEP) as a band of restricted migration.^1^ SPEP is utilised to identify and monitor patients with plasma cell dyscrasias, in particular MM. Immunofixation electrophoresis (IFE) is performed to confirm the presence of the monoclonal protein and to characterise its immunoglobulin heavy chain class and light chain type.^1^ In some patients with a monoclonal gammopathy, SPEP may show a normal pattern or only hypogammaglobulinaemia. The monoclonal protein may also be masked on SPEP if it migrates in the beta or alpha-2 regions. In these patients, an IFE can reveal or exclude the presence of the monoclonal protein.^3^ IFE is a relatively expensive laboratory test requiring greater technologist time and input compared with other chemistry tests that utilise automated platforms. Nevertheless, the practice of routine reflex or reflective IFE testing following the detection of a suspected monoclonal band or other suspicious findings on SPEP or UPEP is commonplace in many laboratories.^4^

Reflex testing refers to the practice of automatic addition of laboratory tests to an existing test request on the basis of laboratory-defined algorithms. Reflective testing refers to tests added on by pathologists or clinical biochemists after consideration of a wider range of information (e.g. demographic data, clinical information, previous results and results of other accompanying tests requested).^5^ For the successful practice of reflective testing, laboratory knowledge of this wider range of information, particularly clinical information, is needed. Reflex testing is based almost exclusively on laboratory results and utilises algorithms that may include some limited clinical information such as demographic data. Reflective testing, on the other hand, depends on the expertise and knowledge of the reviewing pathologist, providing the advantage of incorporating other clinical information that reflex testing does not take into account. The soaring costs of laboratory testing, accompanied by increased demand, result in laboratory management having to perform intense scrutiny of test request practices, including reflex and reflective testing.

**FIGURE 1:**
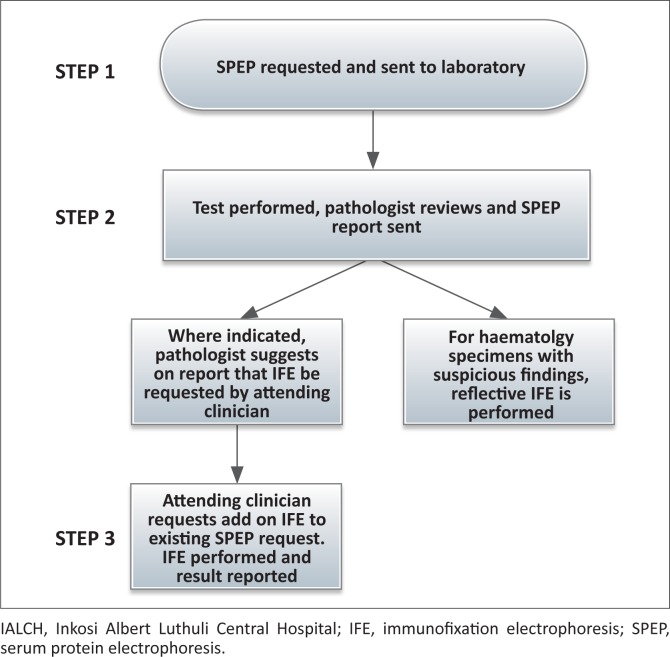
Flow diagram illustrating IALCH laboratory IFE requesting process.

The chemical pathology laboratory at Inkosi Albert Luthuli Central Hospital (IALCH) in Durban, South Africa, is currently the only laboratory that provides SPEP or UPEP services to all state-run healthcare facilities in the province of KwaZulu Natal (estimated population of over 10 million people).^6^ Current practice at the laboratory for all SPEP samples requires that the clinician directly request the IFE in addition to the SPEP (Figure 1). Neither reflex nor reflective IFE is performed routinely by the laboratory; however, following pathologist review, the result report may suggest that the clinician order an IFE for further workup of the patient. Although this above-described practice may reduce costs by preventing unnecessary IFE from being requested by the laboratory, it could result in the delayed diagnosis of a plasma cell dyscrasia, contributing to increased healthcare costs associated with hospitalisations and complications. SPEP and UPEP samples for patients from the hospital‘s clinical haematology department are exempt from the current laboratory IFE policy and reflective addition of IFE occurs where indicated.

Serum-free light chain (SFLC) measurement is a widely-used screening test for plasma cell dyscrasias.^7^ However, this test is still relatively expensive and not offered in many laboratories serving the greater South African population. In addition to abnormal monoclonal proteins, other normal proteins are visualised on SPEP, including proteins that migrate in the alpha-2 and beta regions of the electrophoresis gel. Lakshminarayanan et al. describe 'a two-fold increase in the odds ratio for a positive IFE result when the alpha-2-globulin/ alpha-1-globulin ratio was elevated'.^8^ The authors further recommend reflex IFE testing in patients with a normal SPEP pattern and hypogammaglobulinaemia when an elevated alpha-2- globulin/alpha-1-globulin ratio is detected.^8^

The aims of this study were to determine the number of IFEs requested by clinicians following suggestion by the laboratory and to assess whether the determination of the alpha-2-globulin/alpha-1-globulin ratio, regardless of the presence of hypogammaglobulinaemia, could assist in predicting which of the patients in this population, with no paraprotein detected on SPEP, were more likely to have positive IFE results. A secondary objective was to determine how many SPEP requests were accompanied by clinical histories provided by the clinician or test requestor.

## Research method and design

Data were collected from February 2011 to July 2011 for 1260 consecutive SPEP samples analysed at the IALCH, National Health Laboratory Services chemical pathology laboratory (Durban, South Africa). Samples analysed consisted of routine samples with SPEP orders received by the laboratory from the laboratory hospital and referring hospitals within KwaZulu Natal province.

The Sebia Hydrasys (Sebia, Norcross, GA, USA) automated electrophoresis system was used to perform SPEP and IFE with polyclonal anti-human serum to identify immunoglobulin heavy and light chains.^5^ Quantitation of SPEP fractions was performed using the Sebia Hydrasys densitometer system and Phoresis software (Sebia, Norcross, GA, USA). The same pathologist interpreted all SPEP runs. Requisition by the laboratory of IFE testing was suggested on the result report if a monoclonal band or any other abnormal bands, such as restriction bands (abnormal areas of restriction on protein electrophoresis which may indicate the presence of a paraprotein), were identified on SPEP. IFE testing was also suggested if the SPEP revealed hypogammaglobulinaemia in the absence of a monoclonal band.

The total number of specimens analysed, SPEP and IFE results, patient demographics, clinical histories and requesting clinician details were retrieved from laboratory or hospital information systems. Relevant data from the information systems were analysed and statistical analysis was performed using Microsoft® Excel (Microsoft® Office 2007, Microsoft, USA). A *p*-value of < 0.05 was considered to be of statistical significance.

## Results

**FIGURE 2a:**
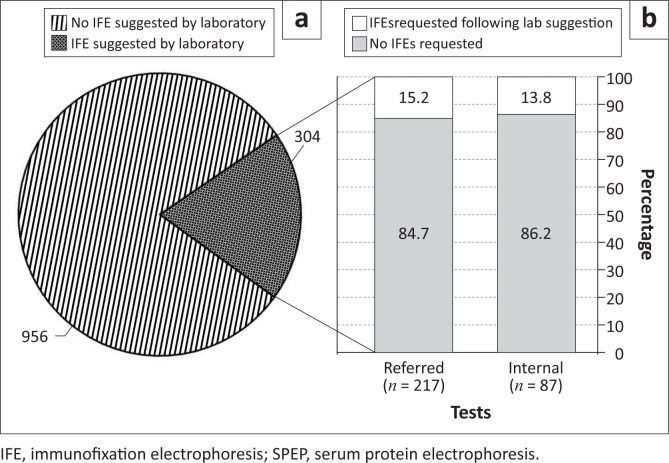
Number of IFE tests (*n* = 304) suggested by the laboratory from among total SPEP requests (*n* = 1260). 2b: Percentage of IFE tests requested following laboratory suggestion for referred (*n* = 33 of 217) and internal (*n* = 12 of 87) samples.

A total of 1260 SPEPs were performed from 1 February 2011 to 31 July 2011. Analysis revealed that the reviewing pathologist suggested 304 IFEs; these comprised 87 internal (IALCH wards and clinics) samples and 217 referred samples (see Figure 2). Fifteen per cent (*n* = 45/304) of the suggested IFEs were requested subsequently by attending clinicians and all IFEs were performed by the laboratory, except for two patients with insufficient samples. Of the 87 internal samples for which IFEs were suggested, 14% (*n* = 12/87) were requested subsequently by the clinicians. Of the 217 referred samples for which IFEs were suggested, 15% (*n* = 33/217) were requested subsequently by the clinician. Almost half (46.5%; *n* = 20/43) of all the suggested IFEs that were performed revealed the presence of a paraprotein, indicating the possible presence of a plasma cell dyscrasia.

Clinical histories were available on the laboratory information system (LIS) for only 53% (*n* = 162/304) of the patients for which IFEs were suggested. Table 1 summarises the clinical histories (*n* = 13) that were available for all IFEs performed (*n* = 43). Of the six positive IFEs for which clinical histories were supplied, four had the clinical diagnosis of 'query myeloma', one was peripheral neuropathy and one was spinal tuberculosis.

**TABLE 1:** Clinical histories for the 43 laboratory-suggested IFE tests requested by clinicians and performed.

Clinical history (as per laboratory information system/requisition form)	Number (*n*)
Query myeloma	6
Neurological complaints (for example: peripheral neuropathy; lower limb weakness)	4
Vertebral collapse	1
Cancer metastases	1
Amyloidosis	1
No information available	30

All the SPEPs resulting in an IFE suggestion by the laboratory were new tests for each of the 304 patients. For 11% (*n* = 33/304) of the patients, a repeat SPEP rather than an IFE was submitted by the attending clinician. The time period between the initial SPEP request and the repeat SPEP ranged from one to 182 days (median 28.5 days). Analysis revealed that of the repeat SPEPs ordered on patients, protein patterns for only three patients changed such that the restriction bands were no longer visualised.

The alpha-2-globulin/alpha-1-globulin ratios were calculated for performed IFEs for patients with and without hypogammaglobulinaemia, as only four of the 43 patients had hypogammaglobulinaemia. The alpha-2-globulin/ alpha-1-globulin ratio mean and standard deviation (SD) for all patients with positive IFEs was 3.2 and 0.9, respectively; for those with negative IFEs, the mean was 3.58 (SD 1.0). Student’s *t*-test revealed no statistically-significant difference between the two groups (*p*-value = 0.2).

## Discussion

This study revealed that only a minority of clinicians request immunofixations following laboratory suggestion for follow-up testing as a result of suspicious SPEP findings. The reasons for this need to be investigated further, but there are several plausible explanations. One explanation may be that not all clinicians, particularly junior clinicians, are familiar with the terms 'paraprotein' and 'immunofixation', or with the implications thereof. Furthermore, clinicians may find it inconvenient to phone the laboratory to request the addition of an IFE test. It must also be noted that requisition of IFE testing was uncommon amongst all clinicians apart from clinical haematologists and haematologist-oncologists. During the study, only two IFEs were requested by general practitioners/clinicians from other specialities.

With regard to the above clinical practices, it is difficult to ascertain the baseline prevalence of monoclonal bands for samples received from the general population. No prevalence data exist for the presence of monoclonal gammopathies in the South African population. Van Vuuren et al. reported a prevalence of 3.2% for monoclonal gammopathies in a group of HIV-infected patients in South Africa.^9^ SPEP, IFE and SFLC have been recommended as screening tests for plasma cell disorders (except primary amyloidosis) by the International Myeloma Working Group. In his study, Katzmann reported evidence to substantiate the use of a simplified screening panel for MM detection, consisting of SPEP and SFLC alone.^10^ However, SFLC studies are not currently available in this setting; furthermore, a large number of patients with MGUS were missed in the study by Katzmann because IFEs were not performed for these individuals.^10^

Worldwide, different studies have shown the prevalence of MGUS to range from 1% to 10% and the prevalence of MM to be around 1% of all cancers diagnosed.^11,12,13,14^ In view of these data, this study’s finding of the presence of monoclonal bands for almost half of those IFEs requested following laboratory suggestion, indicates that the prevalence of monoclonal bands in patients with suspicious SPEP findings is greater than what would be found in the general population. This highlights the need for reflective testing and the addition of reflective immunofixation by the performing laboratory for suspicious findings on SPEP. The results of this study indicate that reflective testing is of particular importance for SPEP requests from clinicians who are not haematologists. This finding is of particular importance because the majority of patients with monoclonal gammopathies present initially to general practitioners and non-specialist clinicians.^15^

Reflective testing requires input such as clinical history in order for a test to be added. The task is made more difficult if clinical histories are not readily available at the time of test review. The findings indicated that clinical histories accompanied only 53% of all SPEPs that had suspicious findings and required possible reflective testing. These findings correlate with another study, which showed that 60% of laboratory request forms did not provide clinical information regarding diagnosis or patient clinical history; however, the availability of clinical information in this study was higher than reported previously in another South African referral laboratory.^16,17^ The lack of clinical information is a significant additional issue affecting interpretation and reflective testing for all laboratory testing. Further education of healthcare workers with regard to inclusion of clinical histories when requesting tests may be needed. This education should ideally start with undergraduate healthcare worker training but should continue as part of continued education and communiqués provided by the laboratory services, particularly to medical interns, phlebotomists and nurses. Another possible method to promote provision of clinical histories could be through utilisation of the laboratory and hospital information system to prevent the complete placement of a test order request if a clinical history is not provided.

In order to assist with the process of reflective testing for SPEP/IFE, the possible use of another parameter, the alpha-2-globulin/alpha-1-globulin ratio, was examined in patients with and without hypogammaglobulinaemia. This parameter is calculated from SPEP densitometry results and thus requires no added cost. The findings in this population did not show any statistically-significant correlation between the ratio and the presence of positive IFE findings. The elevated ratio of alpha-2-globulin/alpha-1-globulin ratio may still have a role in patients with hypogammaglobulinaemia, as described by Lakshminarayanan et al.,^8^ however, the findings could indicate that the ratio is unlikely to assist with detection of a monoclonal protein amongst the population examined in this study.

### Limitations of the study

The use of a single pathologist to report on SPEPs allows for greater consistency. A potential shortfall of this approach is the possibility of overcalling or undercalling of suspicious bands on SPEP by the single pathologist. However, it should be noted that almost half of the suggested IFEs requested were found to be positive. This finding, together with the poor rate of IFE requisition following suggestion, substantiates the need for reflective testing for SPEP.

### Recommendations

Further studies involving immunofixation should be performed for all patients with suspicious SPEP.

### Conclusion

In conclusion, both the laboratory and the user need to ensure that sufficient clinical information is provided to optimise reflective testing in a resource-limited environment.
